# Prevalence and correlates of Vitamin D deficiency in primary school children of South Asian, White European, Black African and Caribbean and White European origin: a cross-sectional survey (2004-2007) in London, Birmingham and Leicester

**DOI:** 10.1017/S0007114525105187

**Published:** 2025-09-23

**Authors:** Angela S Donin, Elizabeth Limb, Jonathan C Y Tang, Peter H Whincup

**Affiliations:** 1 Population Health Research Institute, St George’s, University of London, London, UK; 2 University of East Anglia, Faculty of Medicine and Health Sciences, Norwich, UK; 3 Norfolk and Norwich University Hospitals Foundations Trust Depts of Endocrinology and Clinical Biochemistry, Norwich, UK

**Keywords:** vitamin D, deficiency, children, insufficiency

## Abstract

Vitamin D deficiency is common in the UK, especially in certain ethnic minority populations. There is limited information on childhood vitamin D status in the UK, or the factors associated with vitamin D deficiency. Using a cross-sectional study of 4650 children of South Asian, Black African and Caribbean and White European origins (9-10 years old) surveyed between 2004 and 2007, we investigated measurements of circulating 25(OH)D concentrations (a measure of vitamin D status) and anthropometric measurements. Overall, 68% of children had 25(OH)D concentrations ≤50nmol/L and were either insufficient (25-50nmol/L) (45%) or deficient (<25nmol/L) (23%). Mean 25(OH)D concentrations were lowest in South Asian (especially Bangladeshi) children, intermediate in Black African and Caribbean and highest in White European children. Mean values were ≤50nmol/L for all children during the winter months and ≤50nmol/L throughout the year for South Asian, Black African and Caribbean children. In analyses adjusted for season, age, sex, ethnicity, socio-economic status and fat mass index, girls had a higher risk of being vitamin D deficient or insufficient (OR 1.49, 95%CI 1.32, 1.68) compared to boys. South Asian children (OR 25.49, 95%CI 19.95, 32.57) and Black African and Caribbean children (OR 10.31, 95%CI 10.31, 17.52) had the highest risks of being deficient or insufficient compared to White European children. Childhood vitamin D deficiency was common in this study population. In the UK, targeted and novel interventions are needed to increase 25(OH)D concentrations, particularly South Asian and Black African and Caribbean children and reduce the health risks associated with low vitamin D status.

## Introduction

Vitamin D is a secosteroid hormone with a central role in musculo-skeletal health through its regulatory actions on calcium and phosphorus absorption; adequate vitamin D concentrations are essential to maintain healthy bones, muscles and teeth^(1)^. Vitamin D is an umbrella term for two compounds; vitamin D3 (cholecalciferol) and vitamin D2 (ergocalciferol). Vitamin D3 primarily obtained through endogenous synthesis in the skin with exposure to sunlight (UVB radiation), and to a lesser extent through limited dietary sources including oily fish, eggs and fortified foods. Vitamin D2 can only be obtained from plant-based dietary sources such as mushrooms or fortified foods, and comprises a much smaller proportion of total vitamin D. Following absorption in the small intestine, these vitamins are then converted in the liver to 25-hydroxyvitamin D [25(OH)D]; circulating levels of 25(OH)D are used to assess vitamin D status. Within the UK, vitamin D deficiency is defined as having a circulating 25(OH)D <25 nmol/L, and vitamin D insufficiency is defined as concentrations between 25 nmol/L and 50 nmol/L^(2)^.

Within the UK, vitamin D deficiency remains an important public health challenge^(3)^. It is estimated that about 16% of UK adults and nearly 20% of UK children are vitamin D deficient with much higher proportions of the population estimated to have insufficient 25(OH)D concentrations^(4)^. Furthermore, some population groups are at much greater risk of being deficient; vitamin D deficiency is estimated to affect half of adults of South Asian origin and about a third of adults of Black African Caribbean origin^5,6^), adults from lower socio-economic groups are also at a greater risk of being deficient^(7)^. Despite recommendations from the UK’s Scientific Advisory Committee on Nutrition (SACN) for all adults and children to take a vitamin D supplement of 10 µg in the winter months and at-risk individuals to take supplements throughout the year (including ethnic minority groups with dark skin tones, people who are housebound or who cover their skin when outdoors), vitamin D deficiency remains very common^(3)^. This indicates a lack of adherence to supplementation guidelines, as around 17% of adults are estimated to regularly take supplements ^(4)^. The persistent poor vitamin D status in the UK has led the UK Government to prioritise the development of strategies to improve vitamin D status of the UK population^(8)^.

The high prevalence of vitamin D deficiency is concerning, considering the potential impact on bone health, in addition to the many other immunological and metabolic effects which low circulating 25(OH)D concentrations may have^(9)^. Observational studies report strong associations between vitamin D deficiency and increased risk of infections^(10,11)^, chronic disease risk^(12)^ and mortality^(13,14)^. This evidence highlights the urgent need to investigate and address vitamin D deficiency in the UK population, and particularly populations at high risk ^(3)^.

There is little information on the prevalence and patterns of vitamin D deficiency in children in the UK, although reports from Primary Care records indicate marked increases in the diagnosis of acute vitamin D deficiency in children during the last decade^(15,16)^. Trend analysis of the National Diet and Nutrition survey (NDNS) data suggests that since 2008, average (25(OH)D concentrations in children have decreased, with the latest data indicating that 19% of mainly white European, 11–18-year-olds study population are vitamin D deficient. In younger children there is no clear time trend, with mean concentrations fluctuating over the same period; this may be due to the much smaller samples of this age group providing blood samples^(4,17)^. In addition, there is very limited national UK data on 25(OH)D concentrations in children from ethnic minority groups, who have had limited representation within the NDNS samples (<5%). Using data from a large cross-sectional survey in children of Black African and Caribbean, South Asian and White European origins, we report on the prevalence of vitamin D deficiency and insufficiency in children throughout the calendar year and investigate the determinants of low circulating 25(OH)D in children, including ethnicity, socioeconomic status and adiposity.

## Methods

The Child Heart and Health Study (CHASE) was a large cross-sectional survey of approximately 5000 primary school children of South Asian, Black African and Caribbean, and White European origins. The primary aim was to investigate early risk markers for type 2 diabetes and cardiovascular disease in children of different ethnic origins; methods have been published previously^(18)^. Primary schools in London, Birmingham and Leicester with a high proportion of South Asian children or Black African and Caribbean children were identified and a random sample of 200 schools was recruited; all schools also included between 15-50% white European children to allow ethnic comparisons to be made on a within school basis. Schools were recruited between 2004 and 2007. All year 5 children were invited to participate (aged 9-10 years), ethical approval was obtained from the Multicentre Research Ethics Committee (Wales) and the study was carried out in accordance with the principles of the Declaration of Helsinki.

### Measurements

All measurements were taken between October 2004 and February 2007, during school term time only (including all months except August) by a single survey team, which included three trained research nurses. Measurements of height, weight and bioelectrical impedance (Bodystat Ltd) were recorded for each child and fat mass index was calculated, derived from bioelectrical impedance; a valid measure of body fat in this multi-ethnic population^(19)^.

#### Plasma Vitamin D metabolites

Children provided a fasting blood sample following an overnight fast. EDTA plasma aliquots were separated by centrifugation (4000 rpm for 10 minutes) and stored at -70°C for between 7-10 years until analysis of circulating plasma 25(OH)D was performed at the Bioanalytical Facility, University of East Anglia (Norwich, UK) and undertaken in Good Clinical and Laboratory Practice conditions using a previously unthawed aliquot. 25(OH)D3 and 25(OH)D2 were measured using liquid chromatography-tandem mass spectrometry (LC-MS/MS) in singles, as previously described^(20)^. The assays were calibrated using standard reference material SRM972a from the National Institute of Science and Technology (NIST), and showed linearity between 0 and 250 nmol/L. The inter/intra-assay coefficient of variation (CV) across the assay range was ≤10% and the lower limit of quantification was 0.1 nmol/L. The assay showed an accuracy bias of ±6.7% against the vitamin D external quality assessment scheme (DEQAS) LC-MS/MS method group mean and met the DEQAS certification requirements. Total vitamin D (25[OH]D) was determined from the sum of 25(OH)D2 and 25(OH)D3.

#### Ethnicity and socio-economic status

To categorise the ethnicity of each child we used self-defined ethnicity for both parents or parental information on the ethnicity of the child. When neither of these information sources were available (∼1%), the parental and grandparental place of origin was used, as defined by the child. Children were classified into four main ethnic groups (“white European,” “black African Caribbean” (including both black African and black Caribbean children), “South Asian,” (including Indian, Pakistani, Bangladeshi and other South Asian origins) and “other” (including children of different ethnic groups and those with dual heritage). Information on parental occupation, provided by children and parents, was used to determine socio-economic status using the National Statistics-Socioeconomic Classification (NS-SEC) ^(21)^. The broad classifications were managerial/professional, intermediate, routine/manual, and economically inactive (referring to people who were currently unemployed, whether or not they were seeking work).

### Statistical analysis

The distribution of 25(OH)D was reasonably normally distributed and did not require log transformation (supplementary Figure 1). Vitamin D status was grouped into replete (>50 nmol/L), insufficient (25-50 nmol/L) or deficient (<25 nmol/L). The odds ratios for being vitamin D insufficient or deficient compared with being replete were estimated using ordered logistic regression with a random effect for school to allow for clustering. Models were adjusted for age, sex, NS-SEC, fat mass index (FMI) and month of measurement. All analyses were carried out in Stata v18.

## Results

Of the 8,641 children invited to take part, consent and agreement were obtained for 5,887 (68% response rate) and 4,650 children had complete measurements, including circulating 25(OH)D concentrations (54% inclusion rate). There were similar numbers of children from each of the main ethnic groups; 1174 Black African and Caribbean children, 1275 South Asian children, 1115 White European children and 1086 children of ‘other’ ethnic origins.

### Participant characteristics and vitamin D status

Table 1 presents mean 25(OH)D concentrations and vitamin D status by participant characteristics. Overall, about a fifth of children were classified as vitamin D deficient (23%), with a higher proportion in girls than boys (26% vs 20%, respectively). Marked differences in vitamin D status by ethnicity were observed; 43% of South Asian children and 28% of Black African and Caribbean children were vitamin D deficient compared to 3% of White European children. The highest prevalence of vitamin D deficiency was observed in UK Bangladeshi children. Higher percentages of white European children were vitamin D replete (25(OH)D >50 nmol/L) (65%), compared to Black African and Caribbean (19%) and South Asian children (11%). Children in the ‘economically inactive’ socio-economic group were more likely to be vitamin D deficient (32%) compared to children in the ‘managerial/professional’ group (19%). There was a slightly higher percentage of children in the highest quartile for Fat Mass Index who were vitamin D deficient or insufficient (25% and 48% respectively) compared to the lowest quartile (20% and 44% respectively). Finally, children who were measured in the summer months (June-July) had the lowest percentage who were vitamin D deficient (20%) compared to 36% of children in the winter months (December-February).

**Figure 1:**
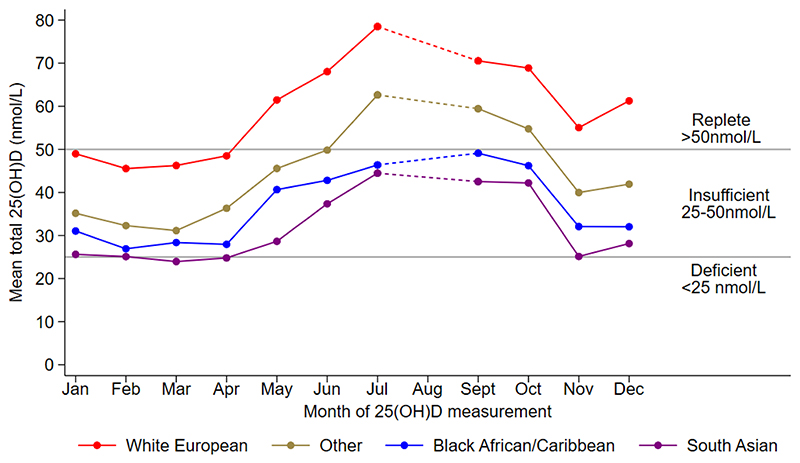
Adjusted mean 25(OH)D by month of measurement and ethnic group Footnote 25(OH)D values are adjusted for sex, age, NS-SEC and fat mass index

**Figure 2:**
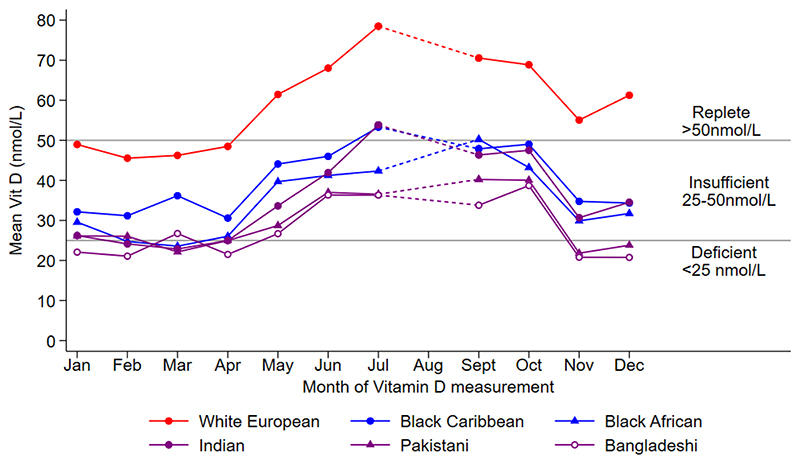
Adjusted mean 25(OH)D by month of measurement and ethnic sub-group Footnote 25(OH)D values are adjusted for sex, age, NS-SEC and fat mass index.

Figure 1 presents the mean 25(OH)D concentrations by month of measurement, separately for each main ethnic group; reference lines representing vitamin D deficiency and insufficiency (see legend) are also included. For White European children, mean 25(OH)D concentrations were within the replete range (>50 nmol/L) for most months measured, and only fell below this between January and April. In contrast, the mean 25(OH)D concentrations for South Asian, Black African and Caribbean children stayed within the vitamin D insufficiency range throughout the year. Figure 2 presents a similar analysis but separates the children further by ethnic subgroup. For most months measured, mean 25(OH)D concentrations for children of Bangladeshi origin were particularly low, being deficient on average in January, February, April and November and insufficient on average for the remaining months of the year. Children of Pakistani origin had mean 25(OH)D concentrations which were deficient in March and November and insufficient for the remaining months of the year.

### Determinants of vitamin D status

Table 2 presents the odds ratios of being vitamin D deficient or insufficient compared to replete by age, sex, ethnicity, socio-economic status and fat mass index, adjusted for season only and then for all the other factors in the analysis. Girls had a higher risk of being vitamin D deficient or insufficient compared to boys (OR 1.45, 95%CI 1.31, 1.61), this risk increased slightly once other covariates were included in the model. South Asian children had a much higher risk of being deficient or insufficient (OR 25.40, 95% CI 19.88, 32.46), and Black African and Caribbean children a smaller increase in risk (OR 13.21, 95% CI 10.18, 17.16) compared to white European children; these higher risks did not alter materially in the model with additional adjustments. The children in the most deprived socio-economic groups had an increased risk of vitamin D deficiency (OR 1.95, 95% CI 1.57, 2.42) which were slightly reduced once other covariates were included in the model. Finally, the risks of vitamin D deficiency or insufficiency increased for each increase in quartile of Fat Mass Index (risk in quartile 4 vs quartile 1; OR 1.24, 95% CI 1.06, 1.45) this association was slightly attenuated once other covariates were included in the model (OR 1.19, 95%CI 1.00, 1.41).

## Discussion

This report presents data on the prevalences of vitamin D deficiency and insufficiency in UK children of Black African and Caribbean, South Asian and White European origins. We found that most children were classified as either vitamin D deficient (26% of girls, and 20% of boys) or insufficient (45% girls and 46% boys). Season, sex, ethnicity, socio-economic status and fat mass index were all associated with vitamin D status with ethnicity being the strongest determinant. South Asian children (particularly Bangladeshi children) had the highest risks of being vitamin D deficient compared to White European children; 89% of South Asian children were either deficient (43%) or insufficient (46%), and 81% of Black African and Caribbean children were either deficient (28%) or insufficient (53%).

The high proportions of children we report who were vitamin D deficient is consistent with adult and adolescent data across Europe, with vitamin D deficiency being described as a pandemic^(22)^. In the present study, vitamin D deficiency is particularly concentrated in ethnic groups with darker skin tones^(22)^. The much higher proportions of children who were classified as having concentrations which were insufficient rather than deficient is also very similar to the proportions reported in European adults, even in sunny climates^(23)^. The associations we report between adiposity and risk of vitamin D deficiency have also previously been reported in both children^(24,25)^ and adults, and suggested to be due to reduced bioavailability of vitamin D in adipose tissue, particularly vitamin D3 which has been synthesised cutaneously following exposure to sunlight^(24)^. This higher risk of deficiency associated with higher body fatness is particularly concerning given the high prevalence of childhood obesity in UK primary school children^(26)^. Similar seasonal patterns and associations with levels of deprivation have been reported previously^(7,27)^.

This large population-based study provides unique data on the vitamin D status of prepubertal children from different ethnic groups within the UK and identifies groups which are at increased risk of deficiency; important to identify for targeted prevention strategies. The design of the study allowed for balanced representation of South Asian children of Indian, Pakistani, and Bangladeshi origin, and of black African and Caribbean children of African and Caribbean origin. Measurements were also taken across all four seasons in sufficiently large numbers to explore seasonal patterns by ethnicity. The overall inclusion rate (54% of children consented and provided complete measurements) is moderate potentially impacting the generalisability of our findings, however similar response rates were seen for each ethnic group and characteristics of respondents were not appreciably different to non-respondents^(18)^. Survey data for this analysis was collected between 2004-2007, and is therefore not current, however it is worth noting that there is a lack of recent data to investigate this multi-ethnic population and that limited data from national surveys do not indicate any substantial changes in either supplement use or vitamin D status in recent years^(4)^. Furthermore, without significant changes to fortification policies or supplementation programmes, experts argue that no change in vitamin D status is likely to occur^(3)^. A further limitation of this survey is that it did not include detailed measurements of bone health such as bone mineral density or parathyroid hormone (PTH), which would have provided important insights into the physiological consequences of vitamin D deficiency in this under-researched and at-risk population. These measures should be included in future studies investigating vitamin D status and bone health in children of ethnic minority origins. Furthermore, we were unable to adequately measure vitamin D supplement use in this population which would be important to explore further along with other determinants of vitamin D status such as amount of sun exposure.

The implications of our findings suggest that vitamin D deficiency is common in UK children, particularly in winter months; for children from ethnic groups with darker skin tones vitamin D deficiency is highly prevalent throughout the year. Bangladeshi children in particular are at high risk of vitamin D deficiency and will need targeted all-year approaches to increase 25(OHD) concentrations. This is consistent with current UK recommendations for high-risk groups, which advocate all-year supplementation. The very low plasma 25(OH)D concentrations suggest that skeletal development may be affected alongside other potential health impacts, such as reduced immunity to infections and increased inflammation^(28–30)^. Evidence also suggests that improving vitamin D status in adults who are deficient may reduce the risk of developing type 2 diabetes^(31)^, which would be particularly relevant to South Asian, African and Caribbean populations who have markedly higher risks of type 2 diabetes than White Europeans^(32)^. Although large RCTs of vitamin D supplementation on type 2 diabetes risk have not yielded consistent declines in type 2 diabetes risk, this may reflect the vitamin D replete status of most trial participants^(33^. Recent evidence suggests a non-linear association between circulating 25(OH)D concentrations and mortality risk, with concentrations below 50nmol/L strongly associated with increased risk^(34)^. This suggests that greatest benefits of vitamin D will be seen for those at the lowest concentrations.

## Conclusion

Vitamin D deficiency in children is a public health concern in the UK. The extremely low concentrations in some ethnic groups need targeted approaches to increase 25(OH)D concentrations and reduce the associated health risks. Novel population-based strategies to improve vitamin D intakes are needed both for the general population and especially for groups at high risk of vitamin D deficiency^(3)^; increasing adherence to supplementation guidelines is one important approach^(35)^.

## Supplementary Material

Supplementary Materials

## Figures and Tables

**Table 1:** Vitamin D status and characteristics of study participants

				Replete (>50nmol/L)		Insufficient (25–50nmol/L)		Deficient (<25nmol/L)		p-value (χ^2^ test)
	N	Mean	(sd)	n	(%) ^1^	n	(%) ^1^	n	(%) ^1^	
**All children**				**145**	**31%**	**2108**	**45%**	**108**	**23%**	
Age (years) ^1^										0.03
Quartile 1	1120	42.4	(21.2)	350	31%	523	47%	247	22%	
Quartile 2	1152	41.9	(21.4)	341	30%	541	47%	270	23%	
Quartile 3	1165	40.5	(21.2)	346	30%	519	45%	300	26%	
Quartile 4	1213	43.5	(22.0)	422	35%	525	43%	266	22%	
Sex										<0.001
Male	2246	44.0	(21.8)	769	34%	1025	46%	452	20%	
Female	2404	40.3	(21.0)	690	29%	1083	45%	631	26%	
Ethnic group										<0.001
White European	1115	59.3	(21.0)	729	65%	358	32%	28	3%	
Black African/Caribbean	1174	36.6	(17.3)	224	19%	625	53%	325	28%	
Black Caribbean	441	40.2	(16.8)	114	26%	243	55%	84	19%	
Black African	648	34.3	(17.6)	97	15%	329	51%	222	34%	
Black other	85	36.0	(14.7)	13	15%	53	62%	19	22%	
South Asian	1275	30.3	(15.5)	140	11%	589	46%	546	43%	
Indian	389	32.4	(16.7)	62	16%	170	44%	157	40%	
Pakistani	464	29.7	(14.5)	41	9%	230	50%	193	42%	
Bangladeshi	318	26.9	(12.6)	17	5%	141	44%	160	50%	
South Asian other	104	35.2	(20.4)	20	19%	48	46%	36	35%	
Other	1086	44.1	(20.2)	366	34%	536	49%	184	17%	
Socio-economic status										<0.001
Managerial/Professional	1242	45.5	(21.9)	476	38%	536	43%	230	19%	
Intermediate	1113	44.4	(22.5)	391	35%	491	44%	231	21%	
Routine & Manual	1252	20.9	(20.5)	360	29%	599	48%	293	23%	
Inactive	777	37.0	(20.0)	173	22%	356	46%	248	32%	
Unclassified/Missing	266	37.1	(19.7)	59	22%	126	47%	81	30%	
Fat mass index (kg/m^5^) ^3^										<0.001
Quartile 1	1163	44.7	(22.7)	412	35%	515	44%	236	20%	
Quartile 2	1162	42.0	(21.5)	378	33%	503	43%	281	24%	
Quartile 3	1163	42.0	(21.4)	358	31%	529	45%	276	24%	
Quartile 4	1162	39.6	(20.0)	311	27%	561	48%	290	25%	
Season measured ^4^										<0.001
Summer (June & July)	810	54.2	(21.9)	428	53%	341	42%	41	5%	
Autumn (Sept–Nov)	1370	46.2	(21.5)	524	38%	625	46%	221	16%	
Winter (Dec–Feb)	1343	34.3	(17.9)	250	19%	611	45%	482	36%	
Spring (March–May)	1127	37.5	(20.0)	257	23%	531	47%	339	30%	

**Footnotes**

1 Percentages sum to 100% for each row.

2 Ranges for age quartiles: 1=8.9-9.65 yrs (8y 10mo – 9yr 7mo); 2=9.66-9.94 yrs (9y 7mo – 9yr 11mo); 3=9.95-10.21 yrs (9y 11mo – 10yr 2mo); 4=10.22-11.5 yrs (10y 2mo – 11y 5mo). 4 children aged 8 and 12 children aged 11.

3 Fat mass index calculated using height to power 5^ref 19^. Ranges for fat mass index (kg/m^5^): 1=0.238-1.568; 2=1.569-2.022; 3=2.023-2.681; 4=2.682-9.185.

4 No measurements taken in August due to school holidays.

**Table 2:** Odds ratios of being vitamin D insufficient or deficient compared with replete.

	N	Odds ratios (95% confidence interval) of insufficient or deficient compared with replete^1^			
		Adjusted for season of measurement		Mutually adjusted^2^ for other covariates	
		OR	95% CI	OR	95% CI
**Age (years) ^3^**					
Quartile 1	1120	1.00		1.00	
Quartile 2	1152	0.98	(0.83, 1.16)	1.00	(0.83, 1.21)
Quartile 3	1165	1.05	(0.88, 1.24)	1.10	(0.90, 1.35)
Quartile 4	1213	1.03	(0.84, 1.26)	1.06	(0.86, 1.32)
**Sex**					
Males	2246	1.00		1.00	
Females	2404	1.45	(1.31, 1.61)	1.49	(1.32, 1.68)
**Ethnic group**					
White European	1115	1.00		1.00	
Black African/Caribbean	1174	13.21	(10.18, 17.16)	13.44	(10.31, 17.52)
South Asian	1275	25.40	(19.88, 32.46)	25.49	(19.95, 32.57)
Other	1086	5.51	(4.38, 6.95)	5.48	(4.34, 6.91)
**Socio-economic status**					
Managerial & Professional	1242	1.00		1.00	
Intermediate	1113	1.14	(0.96, 1.36)	1.17	(0.97, 1.40)
Routine & Manual	1252	1.37	(1.14, 1.66)	1.30	(1.07, 1.57)
Inactive	777	1.95	(1.57, 2.42)	1.91	(1.54, 2.37)
Unclassified / Missing	266	1.97	(1.50, 2.59)	1.75	(1.30, 2.36)
**Fat mass index (kg/m^5^) ^4^**					
Quartile 1	1163	1.00		1.00	
Quartile 2	1162	1.10	(0.92, 1.31)	1.09	(0.91, 1.31)
Quartile 3	1163	1.17	(0.99, 1.39)	1.12	(0.94, 1.34)
Quartile 4	1162	1.24	(1.06, 1.45)	1.19	(1.00, 1.41)

1 Vitamin D status: Replete >50 nmol/L; Insufficient 25-50 nmol/L; Deficient <25 nmol/L

2 Adjusted odds ratios are mutually adjusted for season of measurement, age, sex, ethnic group, socio-economic status, fat mass index.

3 Ranges for age quartiles: 1=8.9-9.65 yrs (8y 10mo – 9yr 7mo); 2=9.66-9.94 yrs (9y 7mo – 9yr 11mo); 3=9.95-10.21 yrs (9y 11mo – 10yr 2mo); 4=10.22-11.5 yrs (10y 2mo – 11y 5mo). 4 children aged 8 and 12 children aged 11.

4 Fat mass index calculated using height to power 5^ref19^. Ranges for fat mass index (kg/m^5^): 1=0.238-1.568; 2=1.569-2.022; 3=2.023-2.681; 4=2.682-9.185
