# Supplementary material for: Prevalence and correlates of vitamin D deficiency in primary school children of South Asian, White European, Black African and Caribbean and White European origin: a cross-sectional survey (2004–2007) in London, Birmingham and Leicester
Source: Br J Nutr. 2025 Sep 23;134(8):689–95. doi: 10.1017/S0007114525105187 (PMC7618223; doi:10.1017/S0007114525105187)
Supplement: Donin et al. supplementary material 1 — Donin et al. supplementary material [file S0007114525105187sup001.docx]

**Supplementary Figure 1. Distribution of 25(OH)D concentrations**
