# Supplementary material for: Prevalence and correlates of Vitamin D deficiency in primary school children of South Asian, White European, Black African and Caribbean and White European origin: a cross-sectional survey (2004-2007) in London, Birmingham and Leicester
Source: Br J Nutr. Author manuscript; Available in PMC 2025 Oct 10. (PMC7618223; doi:10.1017/S0007114525105187)
Supplement: Supplementary file 1 [file EMS208814-supplement-Supplementary_Materials.pdf]

Supplementary Figure 1. Distribution of 25(OH)D concentrations

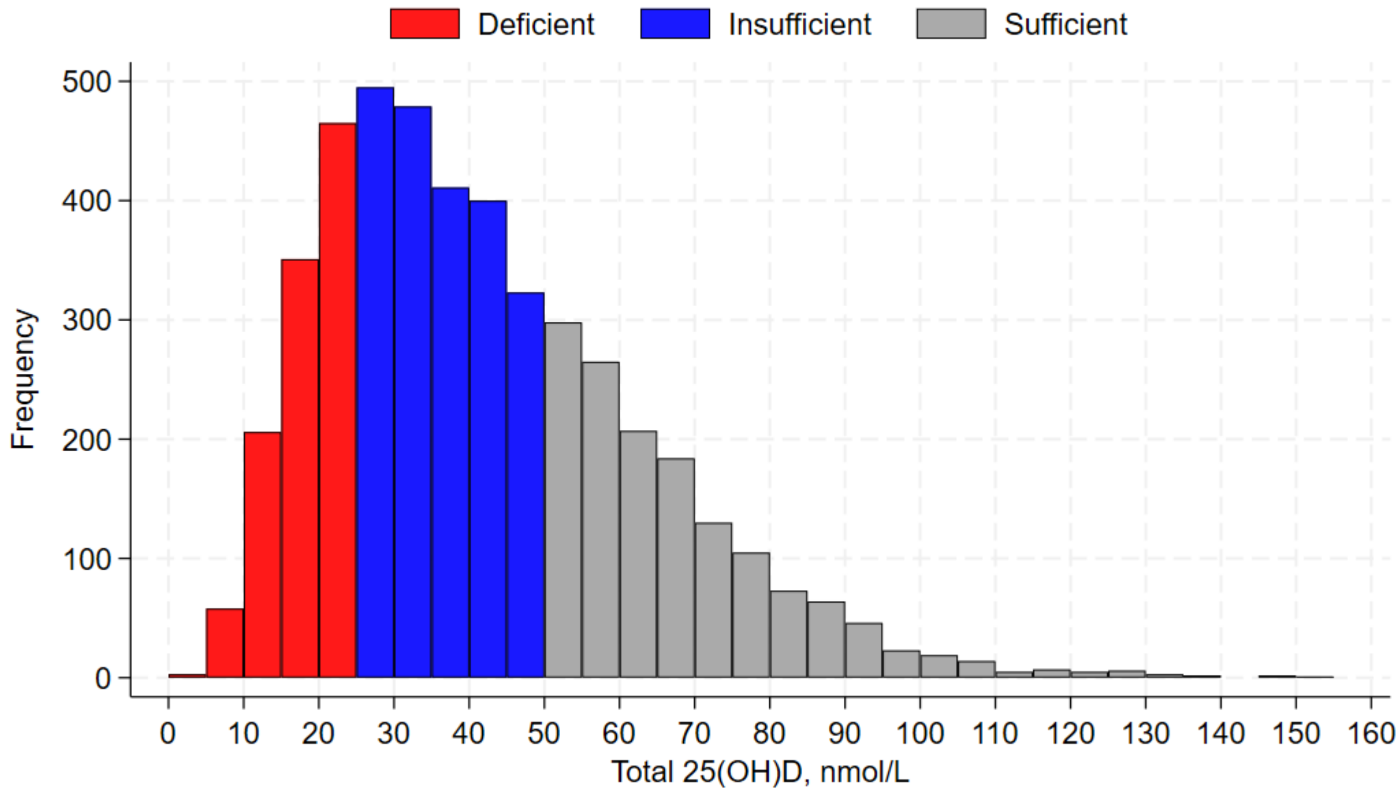
Deficient
Insufficient
Sufficient
Frequency
500
400
300
200
100
0
0
10
20
30
40
50
60
70
80
90
100
110
120
130
140
150
160
Total 25(OH)D, nmol/L
